# Supplementary material for: Association between intraoperative fluid balance, vasopressors and graft complications in liver transplantation: A cohort study
Source: PLoS One. 2021 Jul 9;16(7):e0254455. doi: 10.1371/journal.pone.0254455 (PMC8270449; doi:10.1371/journal.pone.0254455)
Supplement: S2 Appendix — (DOCX) [file pone.0254455.s002.docx]

**SUPPLEMENTARY MATERIAL**

***Association between intraoperative fluid balance, vasopressors and graft complications in liver transplantation: a cohort study***

**S2 Appendix. Supplemental tables and figures**

**S1 Table. Fluid balance and vasopressor use in non-anastomotic strictures**

|  | **No NAS (n=506)** | **NAS (n=56)** |
| --- | --- | --- |
| Fluid balance including ascites(L)^1^ | 0.6 (3.7) | 1.5 (3.9) |
| Fluid balance excluding ascites (L)^2^ | 3.5 (1.7) | 3.7 (2.1) |
| Ascites (L)^3^ | 1.5 [0, 5.0] | 0.5 [0, 4.5] |
| Intraoperative bleeding (L) | 1.0 [0.6, 2.0] | 1.1 [0.8, 2.0] |
| Crystalloid (L) | 4.0 [3.0, 5.0] | 4.0 [3.0, 5.4] |
| Synthetic colloid (L) | 0 [0, 0.5] | 0.5 [0, 0.5] |
| Any vasopressor upon ICU admission | 236 (47%) | 33 (59%) |
| Norepinephrine infusion upon ICU admission^3^ | 224 (44%) | 31 (55%) |
| Vasopressin infusion upon ICU admission | 139 (27%) | 21 (38%) |
| Any inotrope upon ICU admission | 3 (1%) | 0 (0%) |

*Results are reported as number of observed cases (proportion in %) or as means (SD).*

*^1^ 96 missing values in the “no NAS” and 8 missing values in the “NAS” group*

*^2^ 5 missing values in the “no NAS” and 1 missing values in the “NAS” group*

*^3^ 91 missing values in the “no NAS” and 7 missing values in the “NAS” group. Means of the skewed distributions were 2.8 (3.4) and 2.3 (2.9) respectively.*

*Abbreviations: NAS = Non-Anastomotic Strictures, ICU = Intensive Care Unit*

**S2 Table. Any biliary complication – sensitivity analysis with ascites as covariable (multiple imputations)**

| **Variable** | **HR**  **(n = 562)** |
| --- | --- |
| Fluid balance (L) | 0.97 [0.89, 1.07] |
| Ascites | 1.03 [0.97, 1.08] |
| Any vasopressor | 1.04 [0.75, 1.43] |
| Phlebotomy | 0.94 [0.65, 1.37] |
| Age (10 years) | 0.95 [0.82, 1.09] |
| Sex (male) | 0.63 [0.46, 0.87]^*^ |
| MELD | 0.99 [0.97, 1.01] |
| Sclerosing cholangitis | 1.10 [0.52, 2.35] |
| ALF | 0.66 [0.28, 1.56] |
| Retransplantation | 1.14 [0.58, 2.26] |
| Preoperative RRT | 1.75 [0.95, 3.23] |
| Preoperative creatinine (10 umol/L) | 0.99 [0.97, 1.01] |
| Preoperative hemoglobin (10 g/L) | 1.06 [0.97, 1.15] |
| Baseline CVP (mmHg) | 1.03 [0.99, 1.06] |
| Piggyback | 1.24 [0.64, 2.38] |
| Vena cava clamping time (10 min) | 1.08 [0.97, 1.19] |
| Hepaticojejunostomy | 0.55 [0.32, 0.96]^*^ |
| DRI | 1.24 [0.99, 1.56] |

*^*^ Statistically significant*

*Results are expressed with 95% confidence intervals.*

*Statistical interaction between fluid balance and presence of vasopressors at ICU admission was not significant.*

*Abbreviations: HR = Hazard Ratio, MELD = Model for End-Stage Liver Disease, ALF = Acute Liver Failure, RRT = Renal Replacement Therapy, DRI = Donor Risk Index, ICU = Intensive Care Unit*

**S3 Table. Any biliary complication – sensitivity analysis with complications up to 3 months (multiple imputations)**

| **Variable** | **HR**  **(n = 562)** |
| --- | --- |
| Fluid balance (L) | 0.98 [0.93, 1.04] |
| Any vasopressor | 0.99 [0.67, 1.40] |
| Phlebotomy | 1.04 [0.69, 1.59] |
| Age (10 years) | 0.91 [0.78, 1.06] |
| Sex (male) | 0.65 [0.45, 0.92]^*^ |
| MELD | 0.98 [0.96, 1.01] |
| Sclerosing cholangitis | 1.52 [0.68, 3.41] |
| ALF | 0.97 [0.42, 2.22] |
| Retransplantation | 1.18 [0.52, 2.70] |
| Preoperative RRT | 1.24 [0.58, 2.67] |
| Preoperative creatinine (10 umol/L) | 0.99 [0.97, 1.02] |
| Preoperative hemoglobin (10 g/L) | 1.04 [0.94, 1.14] |
| Baseline CVP (mmHg) | 1.03 [0.99, 1.06] |
| Piggyback | 1.67 [0.90, 3.10] |
| Vena cava clamping time (10 min) | 1.08 [0.96, 1.21] |
| Hepaticojejunostomy | 0.39 [0.20, 0.77]^*^ |
| DRI | 1.28 [1.01, 1.62]^*^ |

*^*^ Statistically significant*

*Results are expressed with 95% confidence intervals.*

*Statistical interaction between fluid balance and presence of vasopressors at ICU admission was not significant.*

*Abbreviations: HR = Hazard Ratio, MELD = Model for End-Stage Liver Disease, ALF = Acute Liver Failure, RRT = Renal Replacement Therapy, DRI = Donor Risk Index, ICU = Intensive Care Unit*

**S4 Table. Any biliary complication – sensitivity analysis with vasopressors as a continuous variable (multiple imputations)**

| **Variable** | **HR**  **(n = 562)** |
| --- | --- |
| Fluid balance (L) | 0.97 [0.93, 1.02] |
| Vasopressors^1^ | 0.88 [0.45, 1.70] |
| Phlebotomy | 0.94 [0.64, 1.36] |
| Age (10 years) | 0.95 [0.82, 1.10] |
| Sex (male) | 0.63 [0.46, 0.87]^*^ |
| MELD | 0.99 [0.97, 1.01] |
| Sclerosing cholangitis | 1.10 [0.52, 2.33] |
| ALF | 0.64 [0.27, 1.51] |
| Retransplantation | 1.17 [0.58, 2.36] |
| Preoperative RRT | 1.78 [0.97, 3.27] |
| Preoperative creatinine (10 umol/L) | 0.99 [0.97, 1.01] |
| Preoperative hemoglobin (10 g/L) | 1.05 [0.97, 1.15] |
| Baseline CVP (mmHg) | 1.03 [0.99, 1.06] |
| Piggyback | 1.26 [0.66, 2.38] |
| Vena cava clamping time (10 min) | 1.08 [0.98, 1.19] |
| Hepaticojejunostomy | 0.55 [0.32, 0.97]^*^ |
| DRI | 1.24 [0.99, 1.55] |

*^1^as norepinephrine equivalent in ug/kg/min*

*^*^ Statistically significant*

*Results are expressed with 95% confidence intervals.*

*Statistical interaction between fluid balance and presence of vasopressors at ICU admission was not significant.*

*Abbreviations: HR = Hazard Ratio, MELD = Model for End-Stage Liver Disease, ALF = Acute Liver Failure, RRT = Renal Replacement Therapy, DRI = Donor Risk Index, ICU = Intensive Care Unit*

**S5 Table. Non-anastomotic strictures**

| **Variable** | **HR**  **With imputations**  **(n = 562)** | **HR**  **Complete cases**  **(n = 442)** |
| --- | --- | --- |
| Fluid balance (L) | 1.07 [0.97, 1.17] | 1.08 [0.97, 1.19] |
| Any vasopressor | 1.54 [0.82, 2.88] | 1.63 [0.81, 3.27] |
| Phlebotomy | 1.11 [0.61, 2.04] | 1.32 [0.68, 2.57] |
| Age (10 years) | 0.95 [0.74, 1.23] | 1.06 [0.77, 1.45] |
| Sex (male) | 0.88 [0.51, 1.54] | 1.14 [0.60, 2.17] |
| Sclerosing cholangitis | 0.98 [0.39, 2.45] | 1.56 [0.61, 3.98] |
| Retransplantation | 0.54 [0.19, 1.56] | 0.63 [0.18, 2.23] |
| Preoperative RRT | 2.88 [1.27, 6.53]^*^ | 2.97 [1.27, 6.94]^*^ |
| Piggyback | 1.71 [0.66, 4.43] | 1.99 [0.78, 5.11] |
| Vena cava clamping time (10 min) | 0.91 [0.76, 1.08] | 0.93 [0.79 1.09] |
| DRI | 0.99 [0.69, 1.44] | 0.89 [0.59, 1.35] |

*^*^ Statistically significant*

*Results are expressed with 95% confidence intervals.*

*Statistical interaction between fluid balance and presence of vasopressors at ICU admission was not significant.*

*Hepaticojejunostomy was removed from the model because of multicollinearity. Other variables were removed based on clinical decision to reduce number of variables and stabilize the model.*

*Abbreviations: HR = Hazard Ratio, RRT = Renal Replacement Therapy, DRI = Donor Risk Index, ICU = Intensive Care Unit*

**S6 Table. Anastomotic strictures**

| **Variable** | **HR**  **With imputations**  **(n = 562)** | **HR**  **Complete cases**  **(n = 442)** |
| --- | --- | --- |
| Fluid balance (L) | 0.96 [0.92, 1.01] | 0.97 [0.92, 1.04] |
| Any vasopressor | 0.99 [0.71, 1.37] | 0.99 [0.68, 1.44] |
| Phlebotomy | 0.93 [0.62, 1.38] | 0.92 [0.60, 1.41] |
| Age (10 years) | 0.98 [0.84, 1.13] | 0.95 [0.81, 1.11] |
| Sex (male) | 0.64 [0.46, 0.90]^*^ | 0.58 [0.40, 0.85]^*^ |
| MELD | 0.99 [0.97, 1.01] | 1.00 [0.98, 1.03] |
| Sclerosing cholangitis | 0.88 [0.38, 2.01] | 0.61 [0.19, 1.95] |
| ALF | 0.64 [0.24, 1.67] | 0.44 [0.14, 1.43] |
| Retransplantation | 1.30 [0.66, 2.57] | 1.38 [0.60, 3.19] |
| Preoperative RRT | 0.90 [0.36, 2.25] | 0.85 [0.33, 2.18] |
| Preoperative creatinine (10 umol/L) | 0.98 [0.96, 1.01] | 0.98 [0.95, 1.01] |
| Preoperative hemoglobin (10 g/L) | 1.05 [0.96, 1.15] | 1.08 [0.98, 1.19] |
| Baseline CVP (mmHg) | 1.03 [1.00, 1.07]^*^ | 1.02 [0.99, 1.06] |
| Piggyback | 0.96 [0.41, 2.21] | 1.04 [0.44, 2.46] |
| Vena cava clamping time (10 min) | 1.10 [0.99, 1.21] | 1.08 [0.96, 1.22] |
| Hepaticojejunostomy | 0.55 [0.32, 0.92]^*^ | 0.51 [0.26, 0.98]^*^ |
| DRI | 1.21 [0.95, 1.54] | 1.17 [0.89, 1.52] |

*^*^ Statistically significant*

*Results from a model fitted on data sets with multiple imputations.*

*Results are expressed with 95% confidence intervals.*

*Statistical interaction between fluid balance and presence of vasopressors at ICU admission was not significant in either model.*

*Abbreviations: HR = Hazard Ratio, MELD = Model for End-Stage Liver Disease, ALF = Acute Liver Failure, RRT = Renal Replacement Therapy, DRI = Donor Risk Index, ICU = Intensive Care Unit*

**S7 Table. Hepatic artery complications (stenosis or thrombosis)**

| **Variable** | **HR**  **With imputations**  **(n = 562)** | **HR**  **Complete cases**  **(n = 442)** |
| --- | --- | --- |
| Fluid balance (L) | 0.98 [0.91, 1.06] | 0.97 [0.89, 1.06] |
| Any vasopressor | 0.83 [0.50, 1.38] | 0.86 [0.51, 1.44] |
| Phlebotomy | 1.88 [1.06, 3.32]^*^ | 1.68 [0.91, 3.10] |
| Age (10 years) | 0.73 [0.62, 0.85]^*^ | 0.76 [0.63, 0.92]^*^ |
| Sex (male) | 1.08 [0.64, 1.82] | 1.02 [0.59, 1.76] |
| MELD | 0.96 [0.93, 0.99]^*^ | 0.95 [0.91, 0.99]^*^ |
| ALF | 1.82 [0.75, 4.41] | 2.95 [1.17, 7.46]^*^ |
| Retransplantation | 1.83 [1.07, 3.14]^*^ | 1.61 [0.88, 2.97] |
| Preoperative RRT | 3.95 [1.13, 13.20]^*^ | 4.88 [1.87, 12.73]^*^ |
| Preoperative creatinine (10 umol/L) | 0.99 [0.96, 1.04] | --- |
| Preoperative hemoglobin (10 g/L) | 0.98 [0.86, 1.11] | 0.98 [0.86, 1.12] |
| Baseline CVP | 1.01 [0.97, 1.05] | 1.00 [0.96, 1.05] |
| Piggyback | 0.38 [0.10, 1.42] | 0.35 [0.09, 1.40] |
| Vena cava clamping time (10 min) | 1.01 [0.88, 1.16] | 0.99 [0.83, 1.17] |
| DRI | 0.95 [0.64, 1.40] | 0.95 [0.61, 1.49] |

*^*^ Statistically significant*

*Results are expressed with 95% confidence intervals.*

*Statistical interaction between fluid balance and presence of vasopressors at ICU admission was not significant in either model.*

*Sclerosing cholangitis and hepaticojejunostomy were removed based on a clinical decision to reduce number of variables and stabilize the model. Preoperative creatinine concentration was also removed for the same reasons in the complete cases model.*

*Abbreviations: HR = Hazard Ratio, MELD = Model for End-Stage Liver Disease, ALF = Acute Liver Failure, RRT = Renal Replacement Therapy, DRI = Donor Risk Index, ICU = Intensive Care Unit*

**S8 Table. Primary non-function**

| **Variable** | **OR**  **With imputations**  **(n = 562, 18 events)** | **OR**  **Complete cases**  **(n = 442, 14 events)** |
| --- | --- | --- |
| Fluid balance (L) | 1.14 [1.01, 1.29]^*^ | 1.11 [0.97, 1.27] |
| Fluid balance (quadratic term) (L) | 1.03 [1.01, 1.04]^*^ | 1.03 [1.01, 1.04]^*^ |
| Any vasopressor | 0.91 [0.34, 2.46] | 0.58 [0.16, 2.14] |
| DRI | 1.14 [0.61, 2,13] | 1.28 [0.62, 2.65] |

*^*^ Statistically significant*

*Results are expressed with 95% confidence intervals.*

*Statistical interaction between fluid balance and presence of vasopressors at ICU admission was not significant.*

*Estimates did not change with or without DRI adjustments.*

*The effect of fluid balance is (based on the model with imputations): log(OR) = 0.12*fluid balance + 0.03*fluid balance^2^*

*Abbreviations: OR = Odds Ratio, ICU = Intensive Care Unit, P = Probability*

**S1 Figure. Flowchart of patient selection**

**S2 Figure. Kaplan-Meier curve of biliary complications**

**
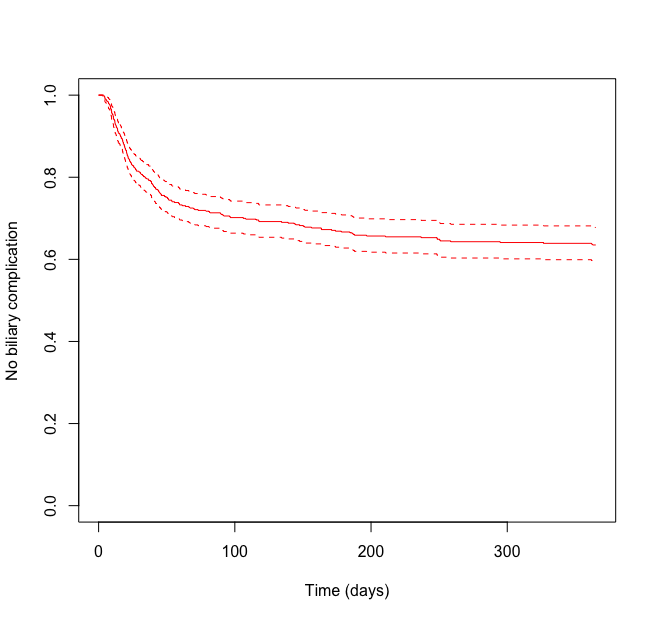
**

*Time to biliary complications expressed as a survival curve. Dotted lines are 95% confidence limits.*

**S3 Figure. Fluid balance and vasopressor doses distribution**

1. **Fluid balance**

**
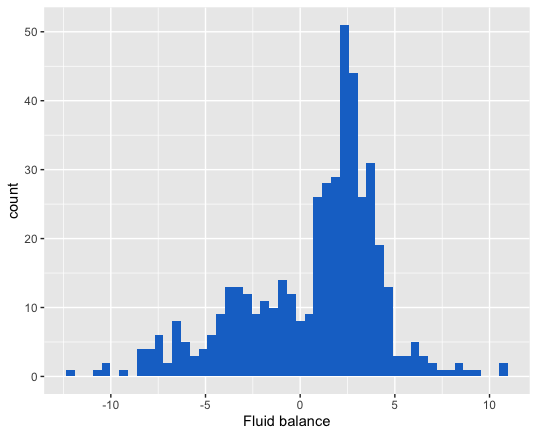
**

1. **Vasopressors doses (among those who had vasopressors)**

**
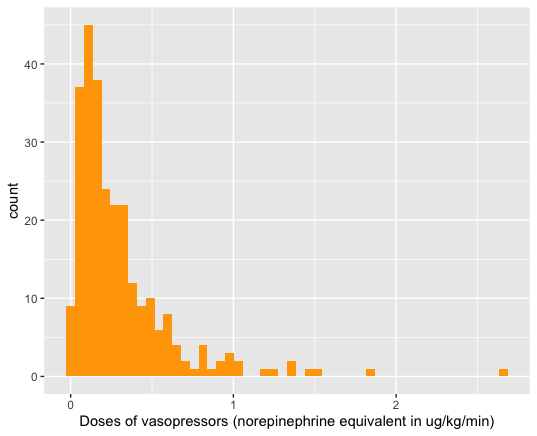
**

*52% of the sample did not have any vasopressor on ICU admission.*

**S4 Figure. Fluid balance according to year of transplantation**

**
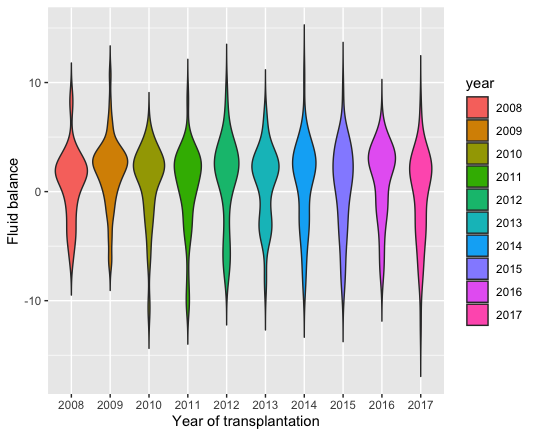
**

**S5 Figure. Fluid balance according to biliary complications**

1. **All biliary complications**

**
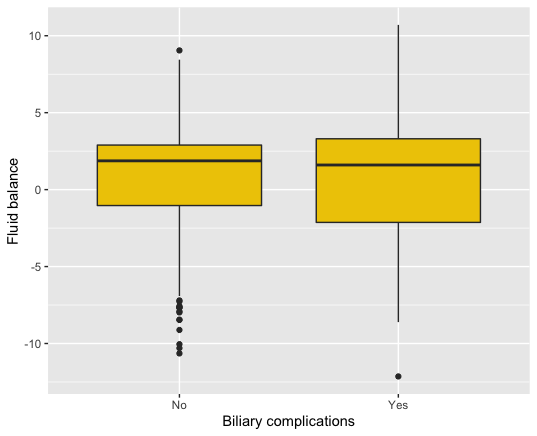
**

1. **Non-anastomotic strictures**

**
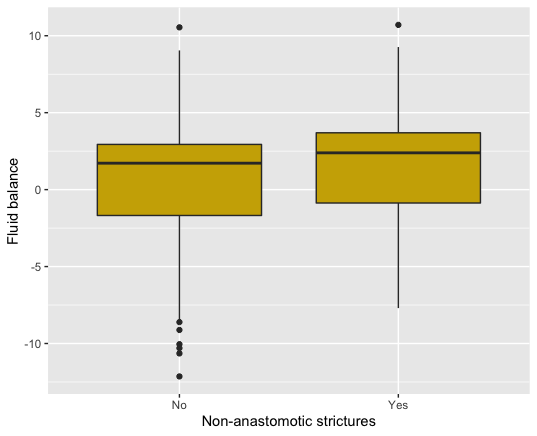
**

1. **Anastomotic strictures**

**
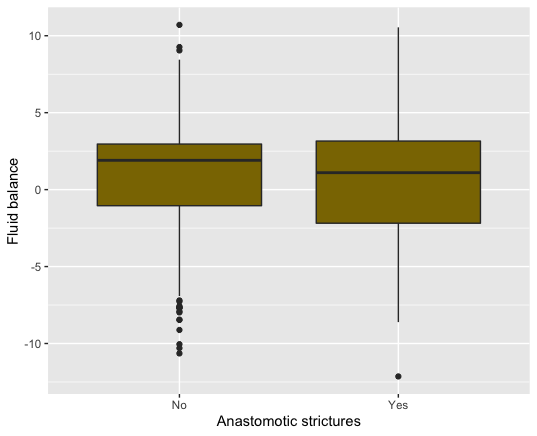
**

**S6 Figure. Doses of norepinephrine equivalent according to biliary complications (among patients who had some vasopressors)**

1. **All biliary complications**

**
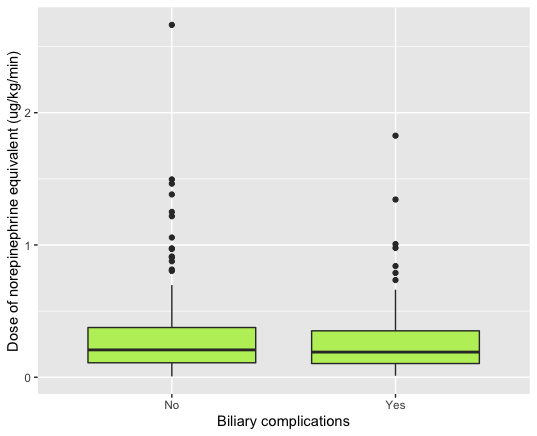
**

1. **Non-anastomotic strictures**

**
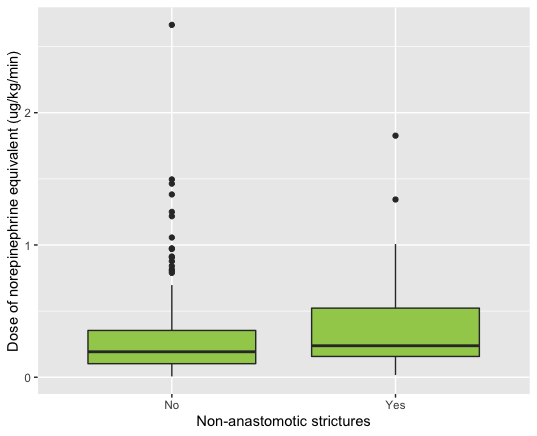
**

1. **Anastomotic strictures**

**
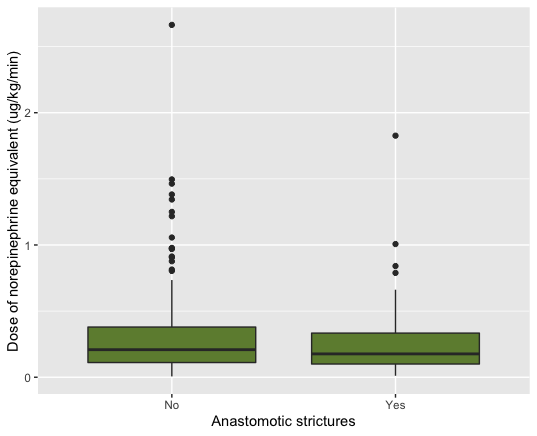
**

**S7 Figure. Odds ratio of fluid balance, according to fluid balance, on primary graft non-function**

**
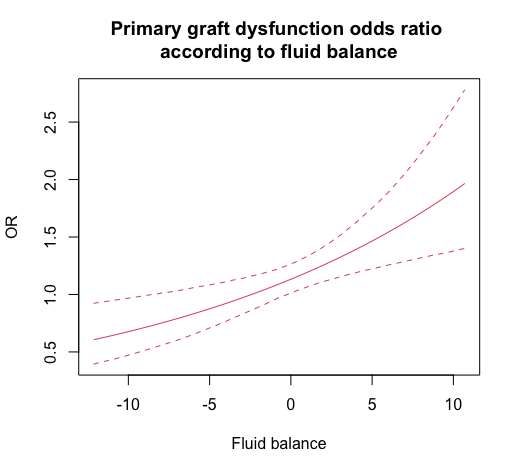
**

*This graph represents the non-linear association between fluid balance and the odds ratio (OR) of primary graft non-function. The main line is the OR according to fluid balance. The association between fluid balance and PNF was non-linear and increased exponentially as fluid balance increased. The dotted lines are 95% confidence limits.*

*OR = Odds Ratio*

**S8 Figure. Survival according to fluid balance**

**
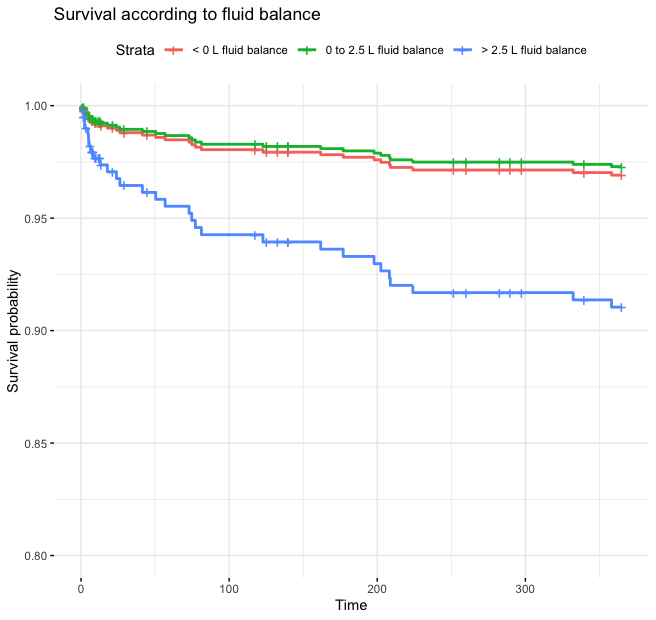
**

*This survival curve was fitted from a marginal multivariable Cox model in the complete cases data set similar to the one reported in table S12 (without the time-dependant coefficient).*

*
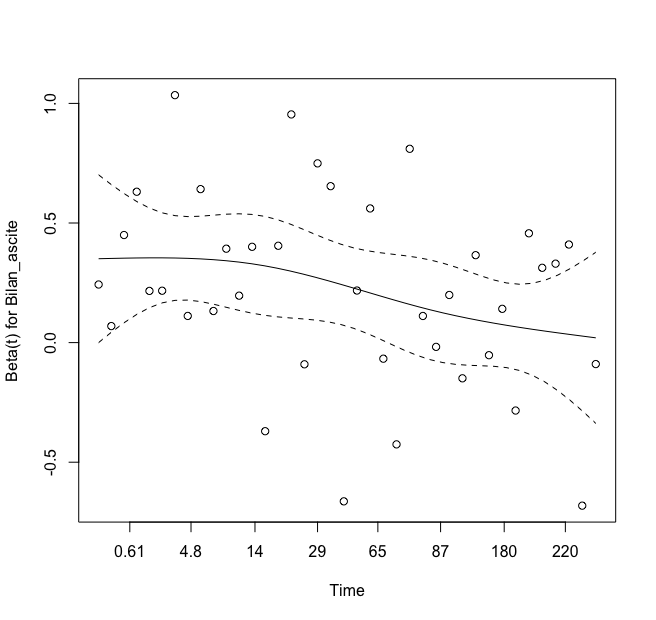
***S9 Figure. Effect of fluid balance over time based on Schoenfeld residuals**

Beta(t) for fluid balance

*A graph of Schoenfeld residuals showing that the Cox regression coefficient for fluid balance decreases over time (and its 95% confidence limits as dotted lines).*
